# Usage of a graph database for the selection of sterile items in the OR

**DOI:** 10.1007/s11548-022-02795-w

**Published:** 2022-11-30

**Authors:** C. Müller, L. Bernhard, D. Wilhelm

**Affiliations:** 1 Research Group MITI, Technical University of Munich, Klinikum rechts der Isar, Munich, Germany; 2 Department of Surgery, Technical University of Munich, Klinikum rechts der Isar, Munich, Germany

**Keywords:** Graph database, User interface, Sterile items, Surgical workflow, Digital operating room

## Abstract

**Purpose:**

In this work, we present a subsystem of a robotic circulating nurse, that produces recommendations for the next supplied sterile item based on incomplete requests from the sterile OR staff, the current situation, predefined knowledge and experience from previous surgeries. We describe a structure to store and query the underlying information in terms of entities and their relationships of varying strength.

**Methods:**

For the implementation, the graph database Neo4j is used as a core component together with its querying language Cypher. We outline a specific structure of nodes and relationships, i.e., a graph. Primarily, it allows to represent entities like surgeons, surgery types and items, as well as their complex interconnectivity. In addition, it enables to match given situations and partial requests in the OR with corresponding subgraphs. The subgraphs provide suitable sterile items and allow to prioritize them according to their utilization frequency.

**Results:**

The graph database was populated with existing data from 854 surgeries describing the intraoperative use of sterile items. A test scenario is evaluated in which a request for “Prolene” is made during a cholecystectomy. The software identifies a specific “Prolene” suture material as the most probable requested sterile item, because of its utilization frequency from over 95%. Other “Prolene” suture materials were used in less than 15% of the cholecystectomies.

**Conclusion:**

We have proposed a graph database for the selection of sterile items in the operating room. The example shows how the partial information from different sources can be easily integrated in a query, leading to an unique result. Eventually, we propose possible enhancements to further improve the quality of the recommendations. In the next step, the recommendations of the software will be evaluated in real time during surgeries.

## Necessity of a non-sterile robotic assistance in the operating room

For the successful workflow of a surgery, the actions and decisions of surgical nurses are of great importance. One of the most significant factors for the quality of the surgical workflow is therefore a lack of nursing personnel with its negative consequences such as stress and work overload [1]. Another cause of increasing stress on the surgical nursing staff is the growing complexity of the surgeries and materials used [2]. The usage of robotic assistance in the operating room (OR) aims to overcome these difficulties and reduce the workload for the staff. As a consequence, the operating room of the future will be shaped by a collaboration between man and machine [3]. The MITI research group at TUM, together with project partners, is therefore developing a robot for the non-sterile operating room area as a part of the AURORA project. This robot will perform subtasks of a circulating nurse, including the supply of sterile items such as gloves and suture material.

## Human–machine interface for a robotic circulating nurse

For the purpose of developing a robotic circulating nurse, the conceptual design of an human–machine interface is necessary as described in [4]. Similar to the human circulating nurse, this interface should collect and process the usually incomplete requests for sterile goods of the sterile OR staff and derive concrete orders for the AURORA robot. To attain a high quality of the recommendations, the context of the request should be included. A robotic assistant must be able to comprehensively recognize the context, since highly efficient verbal communication in the OR is based on omitting information that is obvious from the context [5]. While the human OR staff is able to interpret the context correctly, based on many years of experience [6], it is a difficult but crucial task to impart this skill to the robotic assistance. The use of context in robotic speech control is being investigated in a number of scientific papers. For example, [7] uses the context of speech input to improve the identification of spoken words. [8] investigates how context information can be used to identify verbally requested but not precisely specified objects. In this work, the context will consist of information about surgery type and surgeon.

The goal of this work is to design a subsystem that receives input from three sources: verbal communication, nonverbal communication, and context. The subsystem subsequently generates suitable recommendations for sterile items as output. An important aspect of this task is to not only use predefined knowledge, but to incorporate experience from previous surgeries. The software should prefer certain options by flexibly connecting information and weighting the various relationships between the entities. In this sense, the software can also be understood as an important part of the cognitive architecture of the AURORA robot, giving meaning to the available input and producing reasonable answers.

## Features and advantages of a graph database

The topic is characterized by entities such as sterile items or surgery types on the one hand, and the manifold links between these entities on the other. The ability to store information about the relationship between two entities in a flexible and simple way is therefore important for the task. Using a graph database allows these requirements to be satisfied. It allows to easily model and query complex relationships between many entities [9]. Compared to black box models in the field of deep learning, where the internal logic of the model is hidden to the user [10], graph databases are transparent and enable a natural handling of data [9].

For the implementation, we chose Neo4j due to its wide distribution and numerous interfaces. Within the graph database, nodes can be created containing further information. In this way, any entities can be modeled. An important feature is that edges, i.e., relationships, between nodes can be stored as explicit, independent data sets. In addition, further information can be stored in a relationship by adding properties. Finally, the graph formed by the nodes and relationships can be searched for specific substructures, i.e., subgraphs, which can be described and specified using the Neo4j query language Cypher.

## Design of suitable graph database layout

**Fig. 1:**
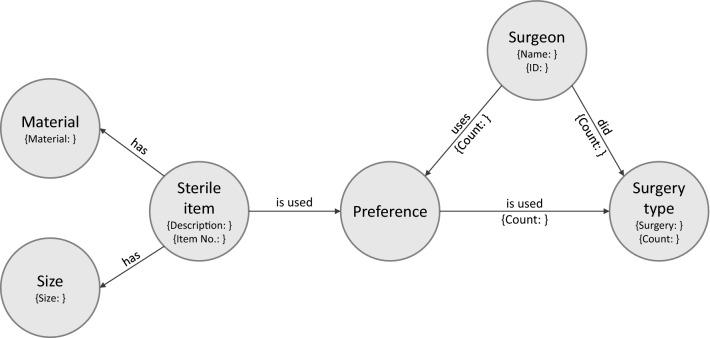
Structure of the graph database with nodes, relationships and particular properties

In this work, a set of entities has been selected to be represented by nodes in the graph. These include surgery type, surgeon and single-use sterile item. An example structure of these nodes and their relationships is shown in Fig. 1. The figure shows how the relationships are used to store which sterile items were used during which surgery types.

To reduce the complexity of the graph, a preference-node is introduced to model an intermediate step between the nodes for sterile item and surgery type. The idea is to establish a surgeon-specific proportional relation to the total number of sterile items used in a surgery type. This is represented by the relationship between the Preference node and Surgeon node. In this way, it is possible to identify the sterile item a surgeon prefers in a specific type of surgery. Thus, the preference-node allows a preference-based assignment of the sterile items in a surgery type.

To prioritize sterile items based on experience from previous surgeries, the relationships store how often they were active in the past. This is done through the property count, which is incremented if the relationship is used. Properties of sterile items were modeled as independent nodes in some cases. This approach is particularly recommended for properties that are often part of search queries. In the present work, this was implemented for the properties size and material.

## Querying the graph database for sterile items that suit the given request and context

The next step is to identify sterile items from the graph database as a suitable recommendations in response to a request from the sterile OR staff and under consideration of the present context. Based on the context, for example, the restriction to a specific surgery type and a specific surgeon is given. In addition, there could be the verbal request for “Prolene” suture material.

The query sent to the graph database via Cypher is a search for certain subgraphs, i.e., for constellations in the entire graph, that fulfill certain structural- and content-related requirements. In this example, it is specified that a matching subgraph must contain the specific nodes of the surgeon, the surgery type, the given material and, in addition, a node of the type sterile item, which has not yet been further specified. These nodes must be related to each other according to the structure shown in Fig. 1. The matching subgraphs returned by the graph database are then evaluated. For each returned subgraph, the description and item number of the contained node of the type sterile item are extracted. The utilization frequency of the sterile item can be derived from the number of uses of the sterile item and the number of completed surgeries, which are also extracted from the subgraph. The utilization frequency is the quotient of the number of uses of the sterile item and the number of completed surgeries. An example for the utilization frequency could be that a queried sterile item was used in 234 out of 255 surgeries, corresponding to an utilization frequency of 92%. Finally, the sterile items from all subgraphs are sorted according to their utilization frequency and thus prioritized.

## Populating and initial testing of the graph database

A graph database was created automatically with records on the sterile item use of 854 surgeries, that were extracted from the clinical information system. These records document for each intervention the surgery type, the surgeon and which sterile items were used. Based on this information, the nodes and relationships of the graph database were created according to the structure shown in Fig. 1. To build the graph database, each surgery record was queried during the import to determine if the required nodes and relationships already exist. If the nodes and relations of an imported dataset already exist, the nodes and relations were incremented in the respective property count. In case individual nodes or relationships of a dataset did not yet exist, additional nodes and relationships were added to the graph database.

For an initial test of the software, it is assumed “Prolene” is requested during a cholecystectomy. “Prolene” is a material used for surgical sutures. A query for this suture material results in seven matching sterile items. For this query, the utilization frequency is evaluated in Table 1 (left). “Prolene 0 FSL 45 cm” was used in 95% of all cholecystectomies, while “Prolene 3/0 FS2 45 cm” was used in 12%. The other five suture materials were each used in less than 3% of surgeries. The utilization frequency of another query, for example, the size “3/0,” commonly used by surgeons to request a suture, is also shown in Table 1 (right). In both test cases, the utilization frequency results in a distinct preference for a specific sterile item, which subsequently could be suggested by the robotic circulating nurse.

**Table 1:** Utilization frequencies of sterile items in % of surgeries they were used in. As input for the software, two different requests “Prolene” (material) and “3/0” (size) were used. The surgery type was set to cholecystectomy (n = 193)

“Prolene”		“3/0”	
Description	Frequency [%]	Description	Frequency [%]
Prolene 0 FSL 45 cm	95	Monocryl 3/0 FS2 45 cm	97
Prolene 3/0 FS2 45 cm	12	Prolene 3/0 FS2 45 cm	12
Prolene 2/0 FS 45 cm	2	Novafil 3/0 V20	3
+ 4 others	< 2	+ 7 others	< 3

## Conclusion

We have proposed a graph database for the selection of sterile items in the operating room. The example above shows how partial information from different sources can be easily integrated, leading to an unique recommendation. By the four enhancements outlined below, the quality of the recommendations could be further increased. These enhancements target the knowledge base upon which recommendations are based. First, in the field of context, surgical phases can be integrated as additional nodes and assigned to sterile items via suitable relationships. Existing classifications of surgical phases, such as those of the Cholec80 [11] or EndoVis [12] dataset, could be used. A context recognition system, as described in [13], could provide the current surgical phase for querying the graph database. Second, in the field of query processing, descriptive keywords can be stored in the sterile item nodes. These could be matched with corresponding additional information from the request of the sterile OR staff. Such a keyword could be a color or a utilization purpose such as “drain suture.” Third, additional properties in the relationships may allow for the integration of preferences, such as those of the surgeon and the hospital. The relationships, and thus a sterile item in combination with a particular situation, can be identified as a personal preference or standard operating procedure (SOP). Furthermore, particularly cost-effective or sustainable variants, as well as items with an upcoming expiration date, can be highlighted. These classifications would allow the software to prioritize the items accordingly. [14] uses the idea of storing and using the sterile item preferences of the surgeons for a material management system. Finally, existing entities such as surgery types and sterile items could be enhanced by adding data from other databases like SNOMED CT. SNOMED CT provides descriptions and hierarchies for entities from the clinical context [15]. The terms from these hierarchies could be included as additional labels on the existing nodes. These labels could, for example, enable the identification of nodes as members of higher-level groups.

The next step is to evaluate the inference quality of the software. For this purpose, the software will be used in parallel with a human circulating nurse during surgeries. The recommendations will be evaluated by the surgeon in real time and compared with the sterile items actually supplied by the human circulating nurse.
